# *In Situ* Produced Nanoparticles at the Oil–Water Interface for Conformance Control and Enhanced Oil Recovery

**DOI:** 10.1021/acs.energyfuels.2c01800

**Published:** 2022-10-14

**Authors:** Zhongliang Hu, Layth Al-Ameri, Jabbar Gardy, Mahmoud Alhreez, Dongsheng Wen

**Affiliations:** † School of Chemical and Process Engineering, University of Leeds, LeedsLS2 9JT, U.K.; ‡ School of Aeronautic Science and Engineering, Beihang University, Beijing100191, China; § Shandong Laboratory of Yantai Advanced Materials and Green Manufacturing, Yantai264006, China; ∥ Thi-Qar Refinery, South Refineries Co., Nasiriya64001, Thi-Qar, Iraq; ⊥ Concept Life Sciences Limited, Malvern Panalytical, Unit 69, Listerhills Science Park, Campus Road, BradfordBD7 1h, U.K.

## Abstract

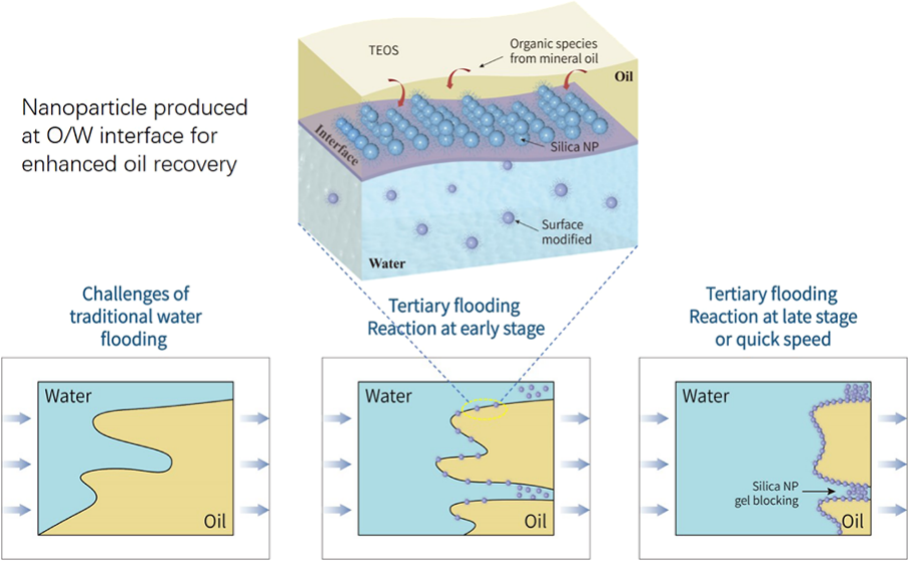

Nanoparticle-assisted enhanced oil recovery (Nano-EOR) has attracted intensive interest in the laboratory as a promising oil recovery technology. However, the nanoparticles’ stability and long-distance delivery of nanoparticles (NPs) in large-scale reservoirs are two main challenges. In this work, we developed a novel concept of *in situ* synthesizing NPs at the oil–water interface inside the reservoir for EOR instead of injecting presynthesized NPs from outside. The pore-scale flooding experiments show that EOR efficiencies for tertiary flooding were 6.3% without reaction (Case 3), 14.6% for slow reaction (Case 1), and 25.4% for relatively quick reaction (Case 4). Examination of the EOR mechanism shows that *in situ* produced SiO_2_ NPs in microchannels could alter the substrate wettability toward neutral wetting. Moreover, the produced NPs tended to assemble on the immiscible oil–water interface, forming a barrier toward interface deformation. As the reaction continued, excessive surface-modified NPs could also diffuse into aqueous brine and accumulate as a soft gel in the flowing path swept by brine. Collectively, these processes induced a “shut-off” effect and diverted displacing fluids to unswept areas, which consequently increased the sweep efficiency and improved the oil recovery efficiency. Auxiliary bulk-scale experiments also showed that the reaction-induced nanoparticle synthesis and assembly at an immiscible interface reduced the interfacial tension and generated an elastic oil–water interface.

## 1 Introduction

It is estimated that the average oil recovery rate from mature oilfields around the world is typically 20–40% of the original oil in place (OOIP) by the conventional oil recovery techniques.^1^ On the other hand, the rate of discovering giant fields has remarkably decreased in the last 2 decades.^2^ Future energy supply will become more reliant on the hydrocarbon produced from nonconventional reservoirs or *via* enhanced oil recovery (EOR) techniques. Recently, injection of nanoparticles (NPs) has been recognized as a promising method for EOR and reservoir characterization, which induced the term Nano-EOR.^3−6^ The deposition or self-assembly of NPs between the oil and solid phase could generate the so-called structural disjoining pressure to facilitate the detachment of oil drop from the solid phase.^7^ They could also alter the wettability of the rock surface and tune the permeability of reservoir rock for better mobility control.^8,9^ Adding NPs can also modify the properties of displacing fluids to improve oil recovery efficiencies, such as generating higher viscosity, proper dielectric properties and conductivity, and lower interfacial tension (IFT) between displacing fluid and the hydrocarbon.^3,4^ NPs for reservoir characterization have also been demonstrated as advanced technology to supplement the traditional method, such as seismic waves whose detection range is in kilometers and logging methods whose detection range is normally in meters.^4^

However, current attempts to use NPs in oil reservoirs are to inject prefabricated NPs from the ground surface deep down to the subsurface.^4^ In order for the NPs to be delivered to the specific location, the NPs have to be stable in the dispersing fluids under reservoir-like conditions and be able to transport a long distance from the injection well to the specific location where the hydrocarbon is trapped. Unfortunately, the NP stability under reservoir-like conditions, namely, complex solution chemistry with high ionic strength, high temperature, and high pressure, presents a big technical challenge to this application. Extensive studies have been undertaken using stabilizers to overcome particle aggregation in reservoir-like environments but with limited success.^10^ These artificial surfactants or polymers are not always good stabilizers because they also suffer from the degradation problem at high ionic strength and temperatures, hence reducing the bonding between NPs and surfactants.^11,12^

The long-distance delivery of NPs in large-scale reservoirs is another main challenge. Generally, increasing ionic strength results in increased NP aggregation and deposition because of the reduced electrostatic repulsion between NPs and solid surfaces.^13,14^ This kind of retention behavior has been observed for many types of NPs including iron,^15,16^ metal oxides,^17,18^ carbon-based particles,^13,19−21^ and quantum dots.^22,23^ However, recent advances in surface coating techniques have enabled stable NP suspensions at high ionic strength (>1 M) with greatly reduced NP attachment in fine-grain porous media (*e.g.*, crushed sandstone).^10,24−26^ The NPs based on oxidized carbon black displayed a good transport ability and stability in field rocks and selective release of hydrophobic compounds when contacting with hydrocarbon, but they still suffered from poor breakthough efficiency at higher temperatures (i.e., >70 °C) and in positively charged dolomite rocks.^27^

The complexity of the natural subsurface environment, especially the multidimensional chemically and physically heterogeneous formation rocks, would also lead to deteriorated NP flow and transport behavior.^28,29^ Phenrat et al. evaluated the transport behavior of olefin-maleic acid copolymer-modified zero-valent iron (nZVI) in a two-dimensional flow cell containing layers of fine, medium, and coarse sand (*d*_50_ = 99, 330, and 880 μm, respectively) with a background electrolyte of 1 mM NaHCO_3_.^30,31^ The authors observed preferential flow of nZVI into regions with higher permeability and deposition in regions of low velocity (low fluid shear), demonstrating the influence of porous media heterogeneity on NP breakthough ability.

Due to these challenges reviewed above, the studies of using *ex situ* produced NPs for EOR still stay at the laboratory scale with many speculative and contradictory results. Instead of injecting prefabricated NPs externally, this study proposes an innovative idea of synthesizing NPs *in situ* at the immiscible oil–water interface inside an unconsolidated core sample to improve oil recovery. This strategy dissolves the reactant in the oil phase and injects ammonia as a catalyst injected along with brine to *in situ* produce and assemble SiO_2_ nanoparticles at the oil–water interface for enhanced oil recovery. In this way, the particle transport problem in harsh reservoir conditions could be sidestepped. The experiments were conducted in a glass microchip serving as surrogate rocks, as well as the flow microreactor. A microchip with a homogeneous structure was selected to reveal the mechanism at the pore scale more easily. Silica NPs were synthesized at the oil–water interface using a sol–gel method. As silica is the main component of the sandstone reservoir, the product does not introduce extra pollution to the reservoir. The reaction rate was indirectly investigated by measuring the interfacial tension at the batch/macroscale before the flooding experiments. The functional group on the surface of the synthesized NP was also checked to understand the surface chemistry of *in situ* synthesized NPs with the influence of the oil phase.

## 2 Materials, Characterization, and Flooding Experiments

### 2.1 Materials

KT24 mineral oil, which is a highly refined mineral oil consisting of saturated aliphatic and alicyclic hydrocarbons, was purchased from Kerax Ltd., U.K., and used as the oil phase. Synthetic American Petroleum Institute (API) brine (nominally containing 8 wt % NaCl and 2 wt % CaCl_2_, laboratory-grade) was used as the formation liquid and the displacing fluid at all flooding stages. Tetraethyl orthosilicate (Sigma Aldrich, U.K.), abbreviated as TEOS, was used as a precursor for silica nanoparticle synthesis. The Sudan blue II dye (Sigma Aldrich) was used to color the oil phase blue. Ammonium hydroxide solution with 28% NH_3_ in H_2_O (≥99.99% trace metal basis) was also purchased from Sigma Aldrich.

### 2.2 Nanoparticle Synthesis at the Oil–Water Interface

**Figure 1:**
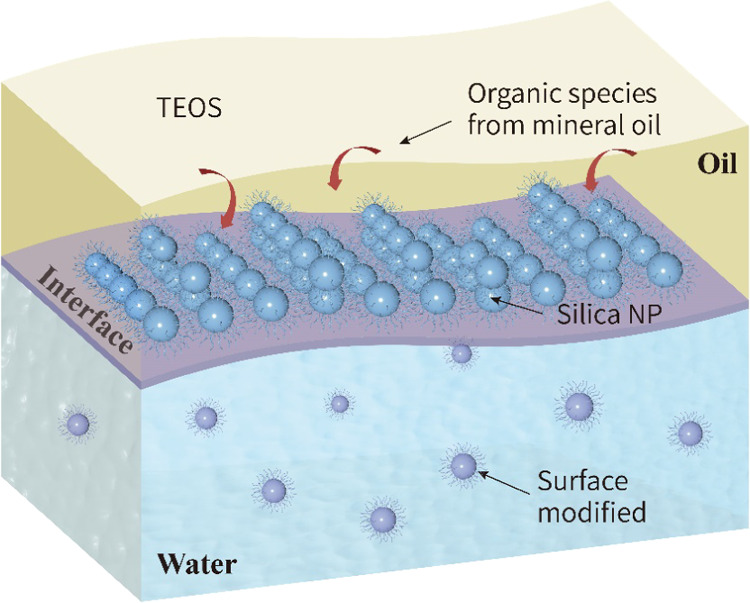
Interfacial reaction and self-assembly of nanomaterials at the liquid–liquid interface.

Silica nanoparticles can be synthesized from the single-reactant TEOS, which was dissolved in the oil phase. The precursor was hydrolyzed and condensed at the oil–water interface to produce SiO_2_ nanoparticles when it diffused to the interface from the oil phase and contacted with brine, as shown in eq 1 and Figure 1. Ammonia was used as a base catalyst to facilitate the hydrolysis process.
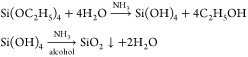
1

Two batches of experiments at bulk scale were performed to examine the influence of ammonia (catalysis) on reaction rate, identify the optimum precursor concentration for the reaction to occur, and provide detailed characterization for the nanoparticle synthesized. For the first batch, various concentrations of TEOS (5, 10, 20, 30, and 50% by volume) were dissolved in mineral oil, and 4 mL of this organic mixture floated on top of API brine to initiate the reaction at the middle interface. While for the second batch, the API brine with 10 vol % NH4OH was used as an aqueous phase to induce NP synthesis and assembly at the oil–water interface.

A relatively high concentration (30 vol %) was applied in the microfluidic experiments to investigate the effect of *in situ* produced nanoparticles on enhanced oil recovery.

### 2.3 Nanoparticle Characterization

A scanning electron microscope (SEM, FEI Quanta 650 FEG-ESEM) operating at 200 kV and an integrated energy-dispersive X-ray (EDX, Oxford X-max 80 SDD) spectroscope with INCA 350 software were used to characterize the morphology and elemental composition of the interfacial synthesized SiO_2_ nanoparticles. The size of the silica nanoparticle in API brine was analyzed *via* a ζ-sizer (ζ-sizer Nano ZS, Malvern Instruments Ltd.) without being extracted from the aqueous phase.

Fourier transform infrared (FTIR) spectra of synthesized silica nanoparticles with and without the presence of mineral oil were obtained at room temperature. An infrared spectrometer (Nicolet iS10, Thermo Scientific) equipped with a diamond attenuated total reflection (ATR) sample cell was used to investigate the chemical vibrational modes of the prepared samples. The spectra were recorded at a wavelength resolution of 4 cm^–1^ in the range of 400–4000 cm^–1^ using 32 scans, where a background spectrum was collected first to cancel out bands from water vapor, CO_2_, and other noise.

The thermal stability of silica nanoparticles was determined using thermogravimetric analysis (TGA) with a TGA/DSC-2 instrument (Mettler Toledo, England). Further, 20 ± 5 mg of the sample was weighed in a 70 μL alumina crucible and placed on the TGA/DSC-2 sample holder. The experiment was conducted under a constant flow rate (50 mL/min) of nitrogen purge gas, and the sample was heated from 30 to 900 °C at 10 °C/min. The thermal behaviors of the synthesized silica NPs with and without the presence of mineral oil were investigated.

The interfacial tension (IFT) between the aqueous phase and the oil phase was measured with a dynamic pendant drop tensiometer (KSV CAM 200). The oil sample was injected by a hooked needle (U-shape) into a quartz cuvette filled with API brine. The volume of the droplet was not controlled, but the injection was stopped just before the oil droplet detached from the needle tip. The values of the IFT were obtained by axisymmetric drop shape analysis. The drop images were recorded using a high-speed camera for 1 h every second. All interfacial measurements were conducted at room temperature and room pressure.

### 2.4 Sample Preparation for FTIR Analysis and TGA

Two types of silica nanoparticles were prepared for the FTIR test. One type was synthesized without the effect of mineral oil by adding 1.2 mL of TEOS in 10 mL of API brine containing 10% NH_4_OH. The sample was then magnetically stirred for 4 h at room temperature for the silica NP synthesis. The other type was synthesized by adding 4 mL of mineral oil dissolved with 30 vol % TEOS into 10 mL of API brine containing 10% NH_4_OH to investigate the effect of oil on the surface component of the synthesized nanoparticles. The NPs were extracted by centrifugation and then sequentially washed with ethanol and deionized (DI) water to remove the remaining organic matter and salt on their surface. The washing process was repeated two to thee times to ensure thorough purification. Finally, the obtained silica NPs were dried in a vacuum oven at 50 °C for 1 day to remove any moisture on their surfaces.

### 2.5 Microfluidic Apparatus

**Figure 2:**
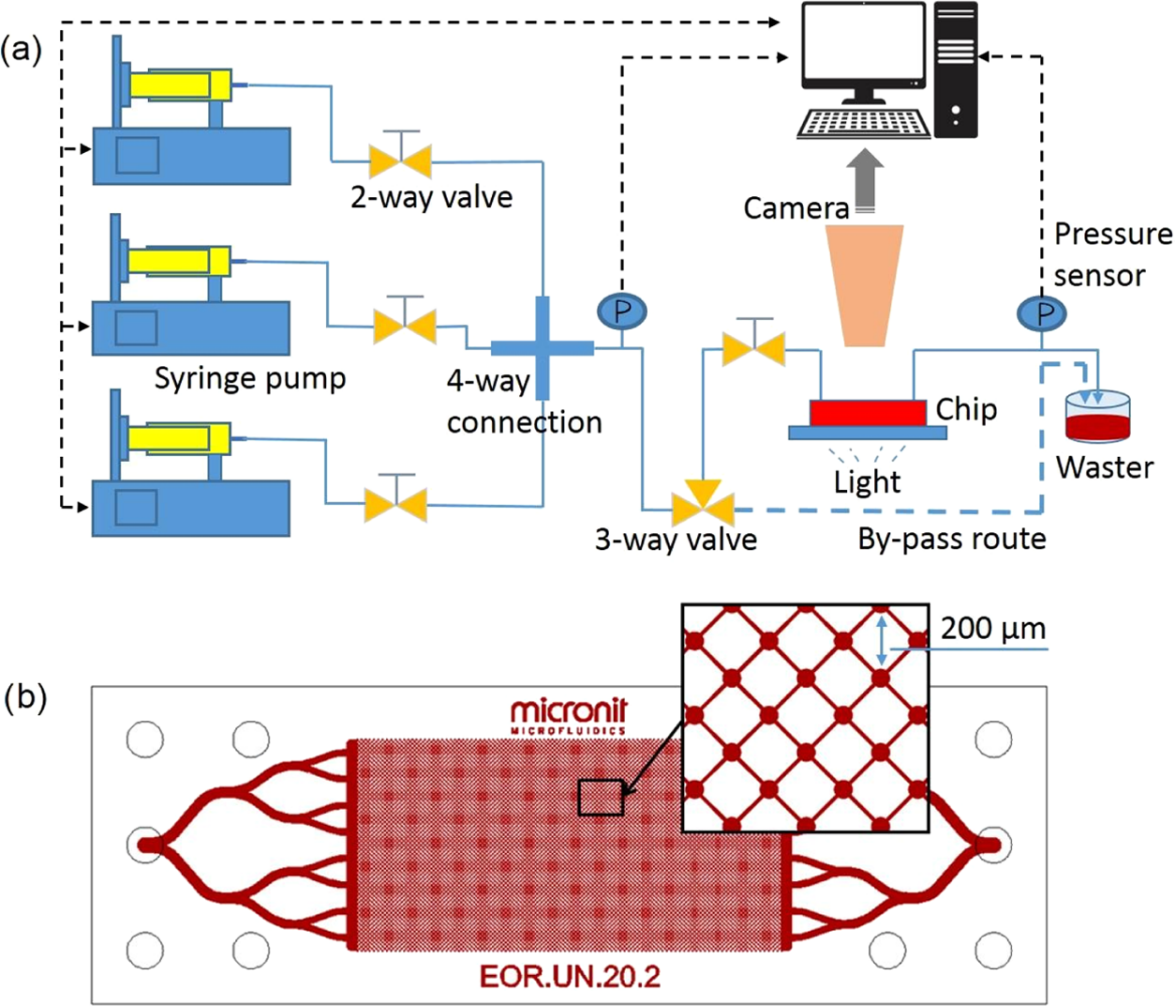
(a) Schematic view of the microfluidic setup and (b) a photograph of the microchip.

In the microfluidic setup, a Nexus 6000 high force infusion syringe pump was used to inject fluids. To avoid interactive contamination, fluids were loaded in thee different syringes separately to deliver brine, oil phase, and displacing chemicals. A schematic diagram of the experimental system is provided in Figure 2a. Flow and transport within the micromodel were visualized using a stereoscopic microscope (SMZ745T, Nikon UK Ltd.) with 7.5× zoom and a 115 mm working distance. The microscope was mounted with a high-speed camera (GS3, Point Grey), which could capture images at a megapixel of 5.0 MP and 2445 × 2048 resolution and record videos at 1080P (full HD) using the Motic Images Plus software package. The pressures were recorded by pressure sensors mounted on the inlet and outlet of the microchip, and the pressure drop was calculated by subtracting the outlet pressure from the inlet pressure.

The displacement experiments were performed in a pillared microchip (45 mm × 15 mm) purchased from Micronite Ltd., the Netherlands. The chip represented a porous medium mimicked by uniformly arranging the arrays of square pillars in a quadrilateral pattern from the entry to the exit of the microchannel, as shown in Figure 2b. The channel and pillars were etched in borosilicate glass. The length and width of the porous area were 20 and 10 mm, respectively. The microchannel was 5 μm wide and etched 20 μm deep, which resulted in a pore area volume of 2.1 μL. If we count the combined volume of the inlet and outlet channels (0.9 μL) and the combined volume of the inlet and outlet holes (2.5 μL), the total internal volume was increased to 5.5 μL. The permeability of the microchannel was determined by flowing water though the microchannel and measuring the resultant pressure drop, and as a result, the permeability of 2.5 Darcy with an accuracy of ±2% was acquired.

### 2.6 Flooding Experiment Procedure

To clarify the influence of *in situ* produced nanoparticles at the oil–water interface, four experiment cases were conducted, as listed in Table 1. For each experiment, the flooding procedure is described as follows.

**Table 1:** Experiment Flooding Cases

	drainage (oil saturation)	water flooding	EOR	chase-water flood
case 1	mineral oil + 30% TEOS	API brine	API brine	API brine
case 2	mineral oil	API brine	API brine	
case 3	mineral oil	API brine	API brine + 10% NH_4_OH	
case 4	mineral oil + 30% TEOS	API brine	API brine + 10% NH_4_OH	API brine

Before the experiments, the mineral oil was colored with 5000 ppm Sudan blue II. The dyed oil was forced to flow though a small syringe filter to remove any dye agglomerates that might block the microchannel. After completing all flooding procedures, the chip was sequentially cleaned with 100 μL of pure acetone, toluene, and isopropyl alcohol (IPA), which was also used for degassing the microchannel, at a flow rate of 100 μL/h for all cleaning solvents. However, when adding TEOS in the oil phase, there could be a hydrolysis reaction for nanoparticle formation at the water–oil interface and probably on the inner surface of the microchannel. Therefore, 0.1 M NaOH solvent was injected into the microchip, which was soaked in a water bath at 50 °C. An ultrasound with an amplitude of 50 was simultaneously introduced by an ultrasonic probe to remove possible particulate matters formed in the channel. Finally, DI water was injected to remove any organic solvent or the 0.1 M NaOH solution after ultrasonication.

**Figure 3:**
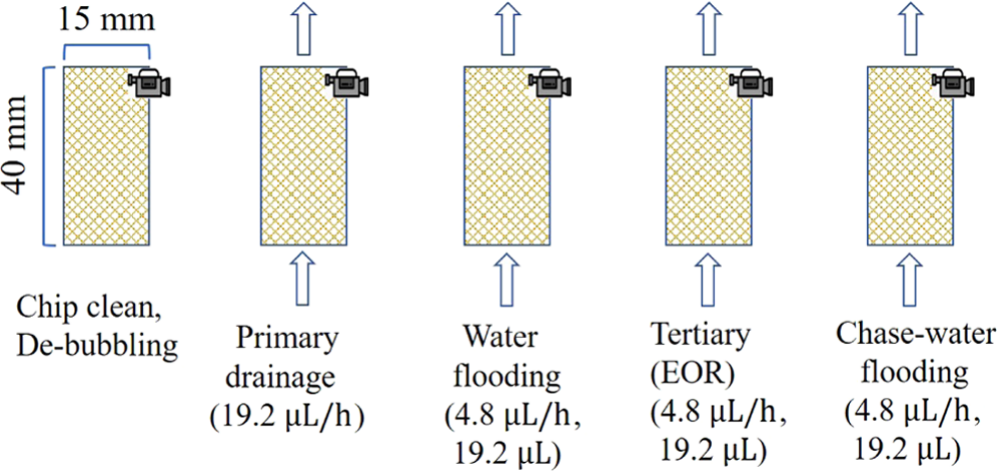
Experiment procedures for each experiment case.

The API brine was injected into the microchip after it was degassed with IPA and then fully saturated with DI water. The experiment was then continued by performing a sequence of primary drainage, water flooding, tertiary flooding (also known as EOR flooding), and chase-water flooding by API brine for all cases using the unsteady-state method, where only one phase was injected into the microchip at a constant flow rate every time (Figure 3). All of the experiments were performed at ambient temperature.

First, the dyed mineral oil was injected into the chip at a flow rate of 19.2 μL/h for the drainage test until the residual brine saturation became constant, while the image of the microchip was recorded every second using a high-speed camera for the subsequent calculation of initial oil saturation, namely, the OOIP. Subsequently, water flooding was conducted by injecting API brine at a flow rate of 4.38 μL/h for a total volume of 19.2 μL, which was equivalent to around 3.5 PV. The oil saturation was recorded and calculated every 2 s.

At the tertiary stage, the API brine containing 10% NH_4_OH was then injected at the flow rate of 4.38 μL/h for 3.5 PV. To trigger the interface reaction and allow time for silica nanoparticle formation, the EOR stage was conducted 1 day after the water flooding. The chase-water flooding was conducted following the EOR stage. To evaluate the long-term effect of *in situ* producing nanoparticles in microreservoirs, chase-water floodings were conducted 2 and 14 days after EOR flooding for Case 4 and Case 1, respectively.

### 2.7 Image Analysis

Image processing software ImageJ was used to quantify the residual oil in the pore and microchannel area. Before being analyzed by ImageJ, all selected images were processed by the Corel PaintShop software using the color “Fade correction” to make them of the same brightness. The probability of different phases (oil phase, brine and glass combined phase) in one of the faded images was then extracted by the “Trainable Weka Segmentation Plugin.” The training result was saved as a classifier model, which was used for all other selected images during the batch processing. The probability maps were then analyzed by a theshold program.

## 3 Results and Discussion

### 3.1 Bulk Interface Characterization

**Figure 4:**
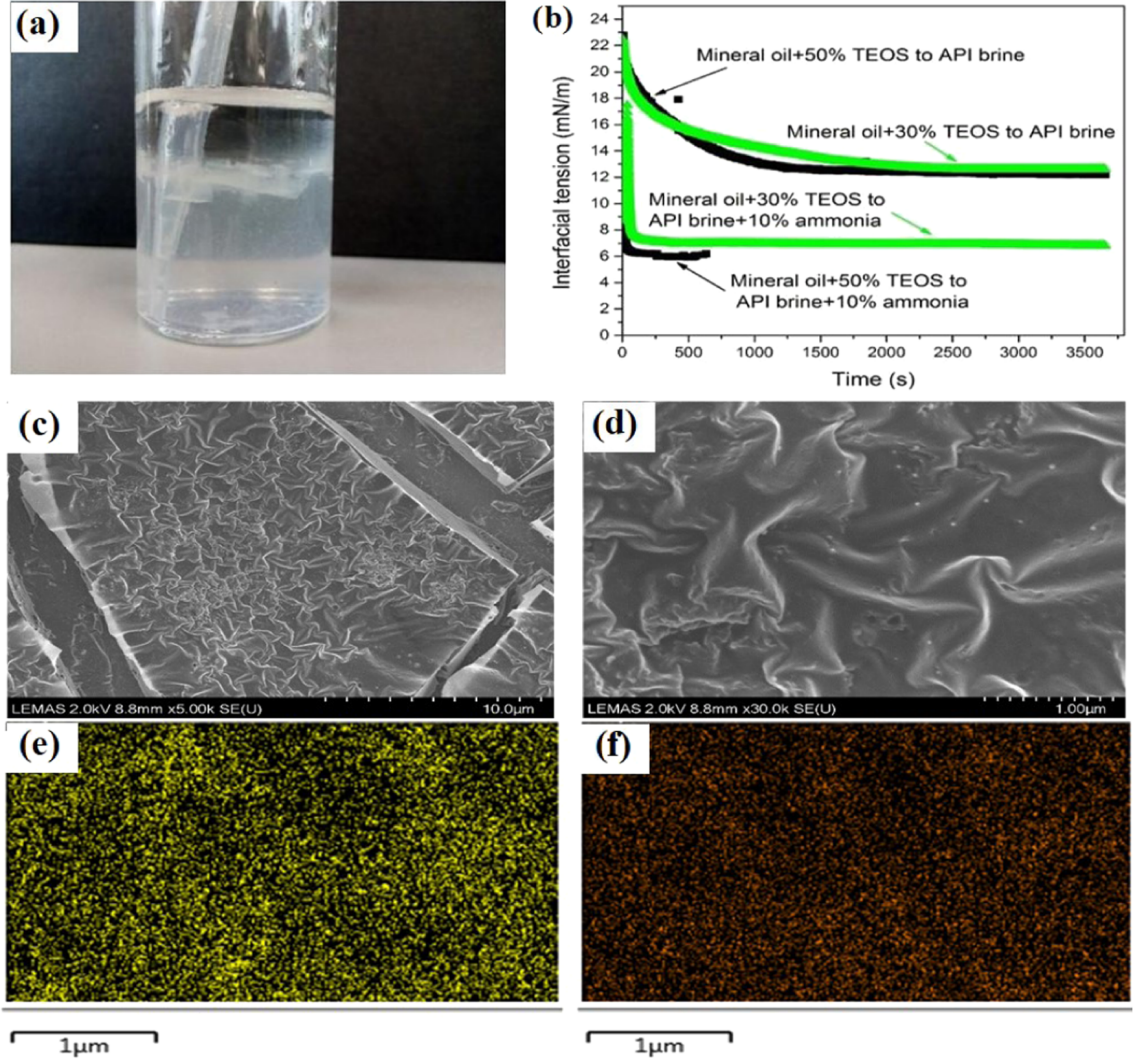
(a) Mashed interface where the silica NPs were formed for the sample containing 5% TEOS in the oil phase and 10% NH_4_OH in the aqueous phase; (b) oil–water IFT changing as the reaction proceeds; (c, d) scanning electron microscopy images for samples extracted from the aqueous phase in (a) on a large scale and on a smaller scale, respectively; (e, f) EDX elemental mapping for (d) for C and Si, respectively.

Constrained by the small volume of the microchannel, it was extremely difficult to *in situ* visualize and characterize the nanoparticle formation at the oil–water interface inside it. However, considering the slow flow velocity in the micromodel for EOR application, it was expected that the nanoparticle formation kinetics would be the same as that in the static macroscale. Figure 4a shows that a silica NP film was formed at the oil–water interface, which could be crushed using a plastic pipette. A silica film was clearly seen from the deformed interface, confirming that the synthesized nanoparticle could stay at the interface after nucleation and growth. The silica NPs were synthesized and then assembled at the oil–water interface as a “nanoparticle surfactant.” Initially, a monolayer of NPs stayed at the interface in a liquidlike manner.^32^ However, as the reaction progressed, NPs were increasingly produced and assembled at the interface and probably on the previously formed monolayer, resulting in a disordered, jammed assembly that could arrest further shape changes of the interface.^33,34^

The IFTs between mineral oil and the API brine are shown in Figure 4b. With the catalysis of ammonia, the IFTs were reduced and an equilibrium state was achieved in only 65 s for both samples containing 30 and 50% TEOS because the hydrolysis reaction quickly occurred at the oil–water interface and the silica NPs were formed there to bridge the oil and water molecules. In contrast, samples without the catalysis of ammonia took a much longer time (>1380 s) to reach the equilibrium state because the reaction rate was slow. Upon adding ammonia, the IFTs between brine and oil containing TEOS were much lower than those without ammonia, regardless of the TEOS concentration of 30 or 50%. The IFT between mineral oil and ammonia was measured as 38.49 ± 0.06 mN/m, which means that ammonia alone cannot reduce the IFT between oil and brine. The IFT recording was uncompleted for 50% TEOS catalyzed by 10% ammonia because the fitting error caused by excessive particle assembly and jamming was too high.

The silica samples for scanning electron microscopy characterization were prepared by extracting the suspension underneath the oil–water interface shown in Figure 4a. It can be seen from Figure 4c that there was a film formed *via* the drying process. There were also some nanoparticles visualized on the surface of the film upon increasing the resolution to 1 μm (Figure 4d). The EDX analysis for the film in Figure 4d verified that the film was a composite material with a high-density carbon element, while the silicon element had a weaker signal but was homogeneously distributed in the film (Figure 4e,f).

### 3.2 Nanoparticle Size and Morphology

**Figure 5:**
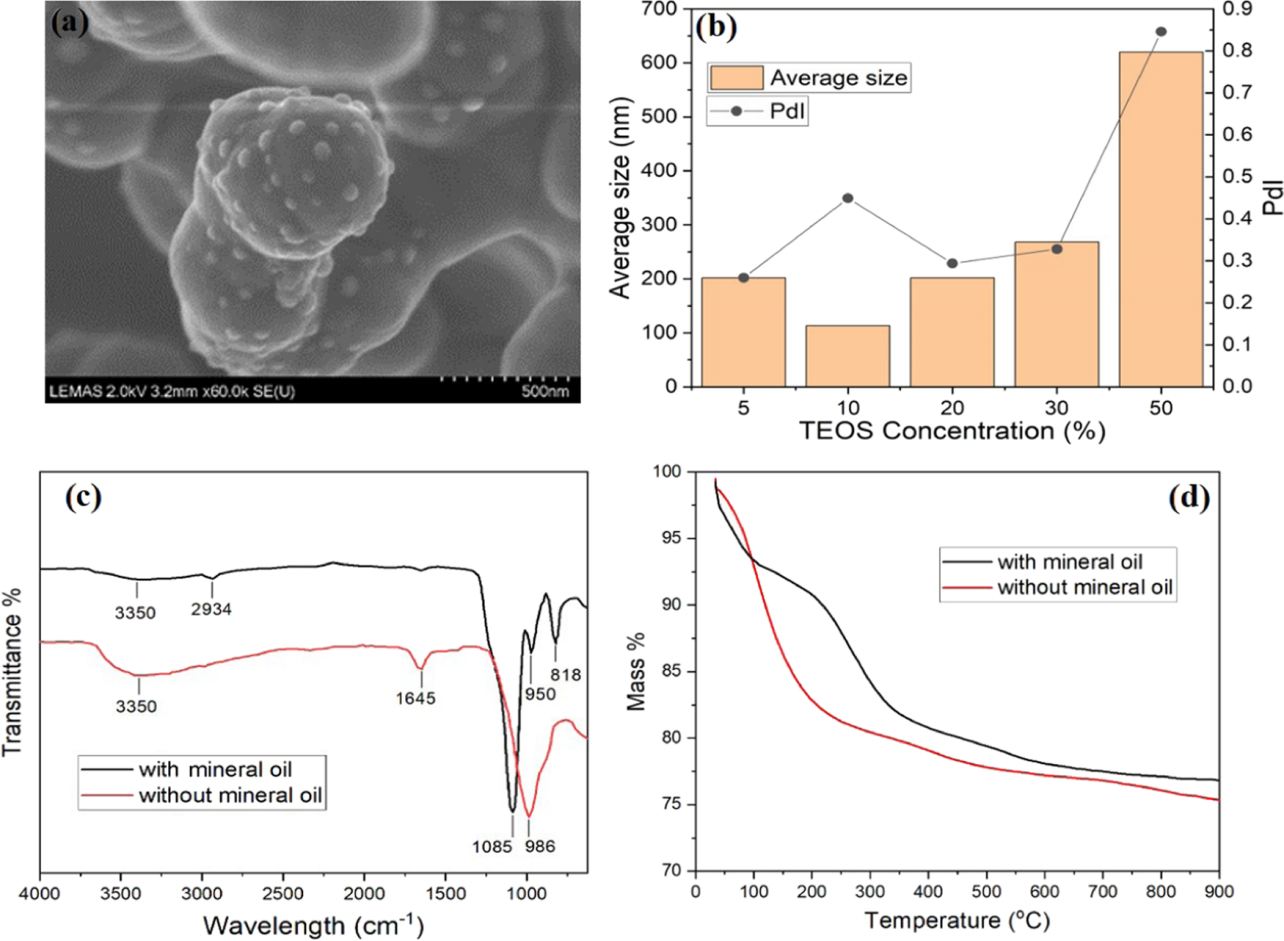
(a) Scanning electron microscopy image for the nanoparticles after aging for 28 days, (b) DLS size distribution of silica nanoparticles in API brine after 15 days of reaction without NH_4_OH, (c) FTIR spectra of produced SiO_2_ nanoparticles, and (d) mass loss profiles measured by TGA.

Figure 4a also shows that there were also extra nanoparticles diffusing into the aqueous phase because the inherent forces (mainly the electrostatic repulsion among NPs) prevented more NPs from adsorbing at the interface once the interface was saturated by a monolayer of NPs.^33^ For scanning electron microscopy sample preparation, the aqueous phase containing dispersed nanoparticles in Figure 4a was extracted and sequentially purified with ethanol and water to remove organic and salt ingredients. Figure 5a shows that the size of the nanoparticles was around 300 nm, and the morphology of the silica nanoparticles can be clearly confirmed as spherical. The small particles (∼50 nm) decorated on the surface of large particles were probably due to a second nucleation or drying process when preparing the scanning electron microscopy samples.^35^ The dynamic light scattering (DLS) data in Figure 5b shows that the hydrodynamic size of silica NPs produced from mineral oil containing TEOS less than 50% was between 100 and 250 nm after 15 days of reaction. The low polydisperse indexes (PDIs) show that the particle size was quite homogeneous. However, 50% TEOS in mineral oil produced NPs with a size of around 600 nm and broad size distribution.

The FTIR spectral analysis shows that abundant silanol groups (Si–OH) at 986 cm^–1^ were detected when directly synthesizing silica without mineral oil,^36^ as seen from the red line in Figure 5c. However, when hydrolyzed from TEOS dissolved in mineral oil, some organic species from the oil phase and TEOS were hypothesized to anchor on the nanoparticle surface. Many of the silanol groups were converted to covalent bonds of Si–O–C groups and Si–O–Si groups, which can be proven by the strong stretching at the broad band of 1085 cm^–1^ for the green line in Figure 5c.^37^ Ballard et al. have found that alkoxylation reaction occurs on silica surfaces and induces a hydrophobic surface.^38^ The mass loss due to decomposition of organic matter between 200 and 350 °C for the red line in Figure 5d also suggests that the silica nanoparticles had been covered with around 10 wt % organic species. The adsorbed organic matters could have a direct impact on the enhanced stability in this environment where the nanoparticle was *in situ* synthesized.

### 3.3 Oil Recovery Efficiency

**Figure 6:**
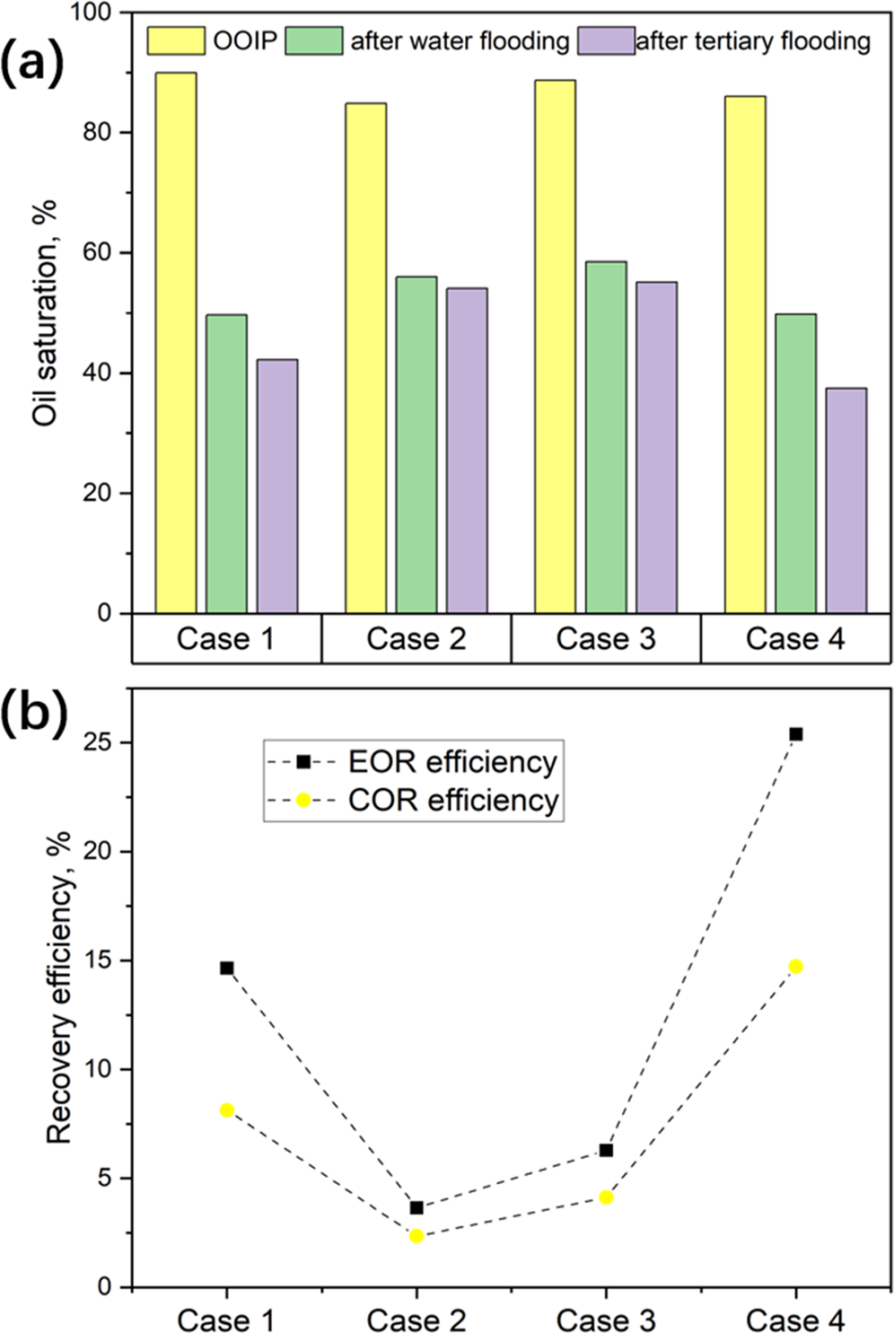
(a) Oil saturation at the end of each flooding stage for different cases and (b) oil recovery efficiency only for the tertiary flooding stage.

We took photos of the oil-saturated area in the microchip regularly on a basis of every 2 s per frame during each flooding stage to subsequently calculate the cumulative oil recovery (COR) efficiency (Figure 6a). The COR efficiency was calculated as the oil saturation at the end of the tertiary flooding stage divided by the initial oil saturation (OOIP) before water flooding. The total COR efficiency for Case 4 (oil + 30% TEOS, 10% NH_4_OH brine) was 56.7%, which means that relative to the OOIP, 14.7% more oil was recovered during the tertiary flooding process (Figure 6b). Standing with the second highest COR efficiency, Case 1 (oil + 30% TEOS, API brine) resulted in 52.7% of the COR efficiency, and it mobilized an additional 8.1% oil during the tertiary flooding stage. With a much lower total COR efficiency of 35.5%, Case 3 (oil, 10% NH_4_OH brine) without TEOS dissolved in the oil phase only resulted in an added COR efficiency of 4.2% relative to OOIP for the tertiary flooding stage, and the lowest additional COR efficiency (2.4%) was found for Case 2 (oil, brine) in which there was also no TEOS added and obviously no reaction-induced oil recovery.

We also paid particular interest to the EOR efficiency that reflects the oil recovery capability specifically for the tertiary flooding stage. Distinguished from the COR efficiency above, the EOR efficiency here was relative to the oil saturation before tertiary flooding or after water flooding. The EOR efficiencies for the stage of tertiary flooding were 14.6, 3.6, 6.3, and 25.4% for Cases 1–4, respectively. For the tertiary flooding, the EOR efficiencies for the cases with TEOS (Cases 1 and 4) were higher than those of the other two cases (Cases 2 and 3), which had no TEOS in the oil phase. The reason for this could be that adding TEOS reduced the IFT between the mineral oil and API brine (Figure 4b) and reduced the viscosity of mineral oil from 42.5 to 35.6 mPa·s. For the tertiary stage, the EOR efficiencies for the cases containing TEOS were still higher than those in the cases without TEOS in the oil because the NPs *in situ* formed at the interface initially behaved as a “nanoparticle surfactant”^39,40^ and then as a conformance control agent as discussed in Section 3.4.2. The produced nanoparticles could lead to lower oil–water IFT, a neutral oil-wetting surface of the microchannel, and higher sweep efficiency, which are specifically explained in Section 3.4. However, if only auditing Cases 1 and 4, the much higher EOR efficiency in Case 4 flooded by brine containing 10% ammonia compared to Case 1 (25.4 vs 14.6%) could be related to the reaction rate. With the catalytic effect of NH_4_OH, silica NPs were more quickly produced at the oil–water interface, resulting in a rapid decrease of IFT in Figure 4b and wettability change in Figure 8.

### 3.4 Pore-Scale Displacement Mechanisms

#### 3.4.1 Wettability Change

**Figure 7:**
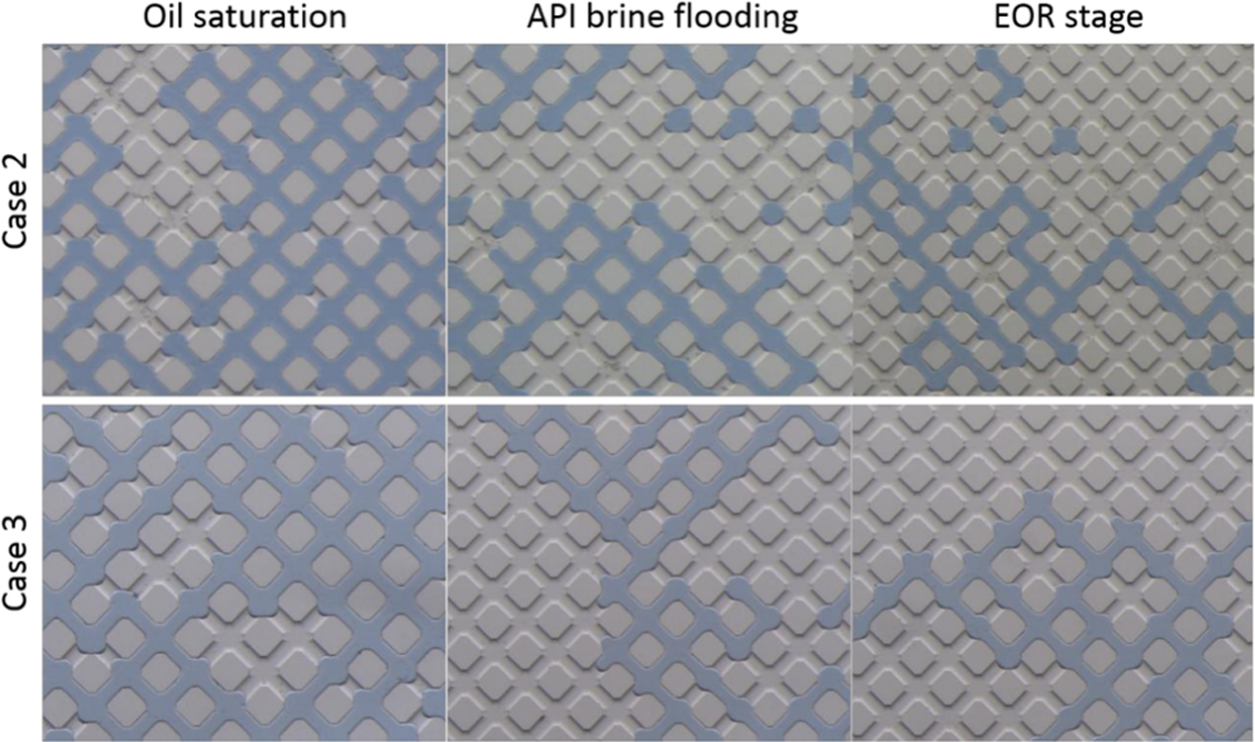
Contact angle at different flooding stages for the cases without TEOS in the oil phase.

**Figure 8:**
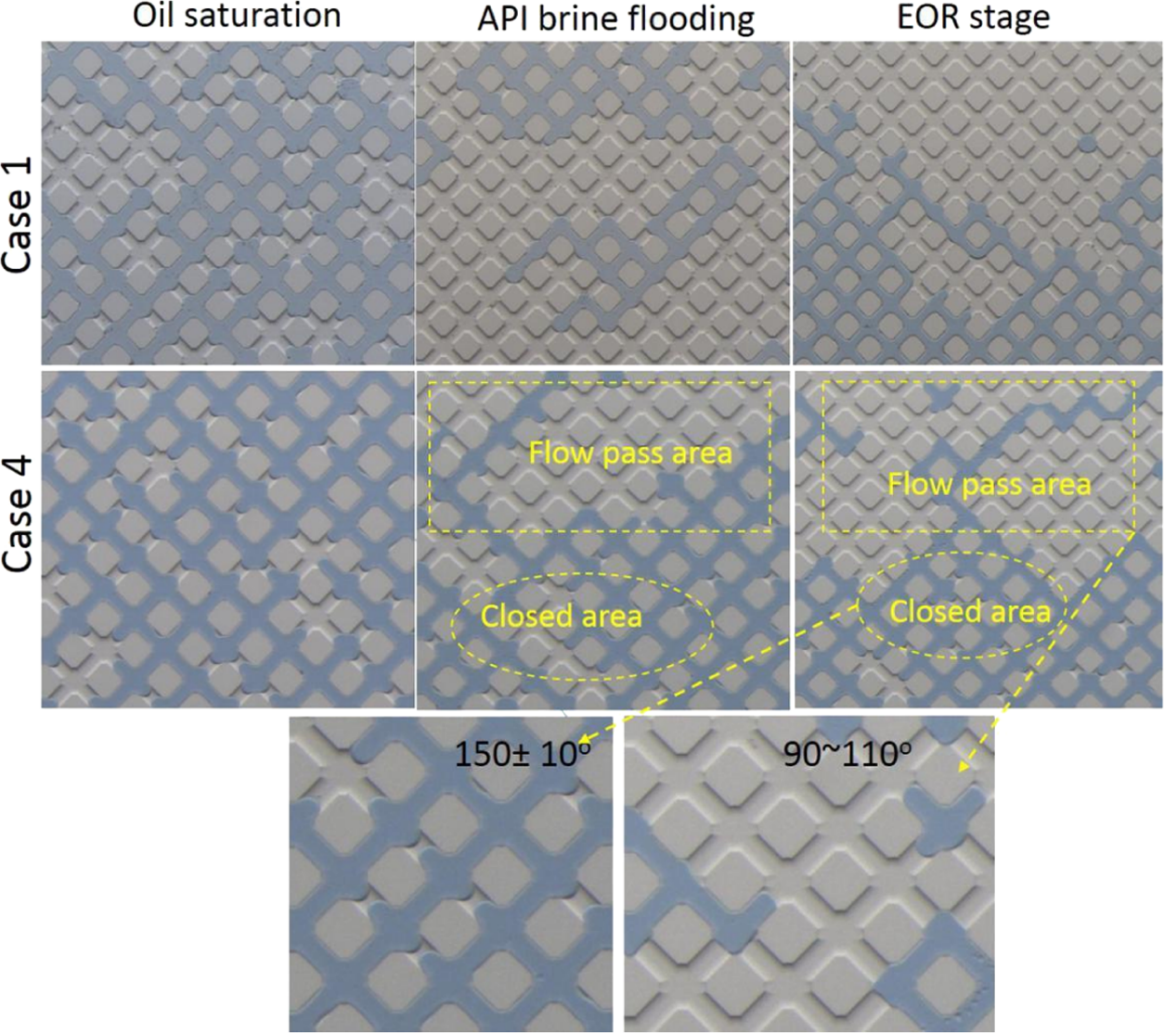
Contact angle at different flooding stages for the cases with TEOS in the oil phase.

Figure 7 shows that without adding TEOS and without the hydrolysis reaction at the interface, the contact angle of the oil phase on the channel surface remained similar thoughout different flooding processes. Figure 8 shows that upon adding TEOS, the contact angle for Cases 1 and 4 was almost unchanged in the oil saturation stage, consistent with the contact angle for Cases 2 and 3. However, the contact angle started to change during the water flooding stage for the cases with precursor (Cases 1 and 4). Upon adding 10% NH_4_OH in the displacing fluid in Case 4, only the contact angles for areas swept by brine containing ammonia (*i.e.*, the area circled by the yellow rectangle in Figure 8) were reduced obviously. In contrast, the contact angle did not change for the closed area, which was not swept by displacing fluids (*i.e.*, the area circled by the yellow ellipse in Figure 8). Overall, the angle wetted by the oil phase in the microchannel was changed toward intermedium at around 90–110° partly because the IFT was reduced by nanoparticles’ assembly at the oil–water interface induced by reaction.^39^

However, based on the Young–Laplace equation, reducing the IFT with unchanged solid–liquid surface tensions will result in a more water-wet micromodel. Therefore, the change toward neutral wetting was also due to the adsorption of organically modified NPs on the microchannel surface. It has been recognized that alkoxylation reaction occurs on silica surfaces, and the surface can be modified toward hydrophobicity.^38^ Luo et al. further provided direct evidence to confirm the hydrophobicity of the alkylated surface by measuring the contact angle on flat silica.^41^ Therefore, it is also believed that the deposition of silica nanoparticles on the inner surface was the main reason for the wettability alteration toward neutral wetting. A previous study also showed that weakly neutral wetting rock could produce optimum oil recoveries,^42^ which is in agreement with our observations.

#### 3.4.2 Conformance Control

**Figure 9:**
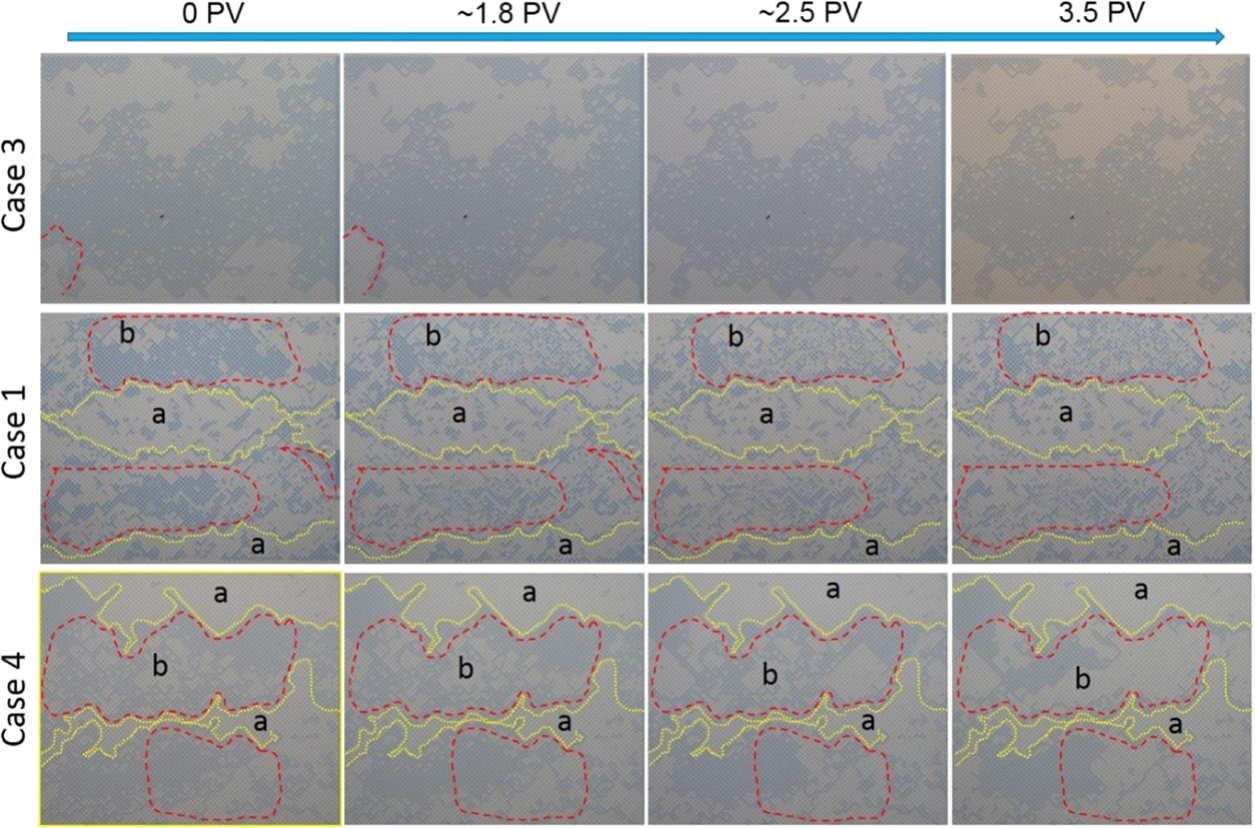
Sequence of oil saturation during the EOR stage for Cases 1, 3, and 4. The area “*a*” circled by the yellow dashed line is the shut-off area, while the area “*b*” enclosed by the red line is the new displaced area.

For the EOR stage, the pore-scale images at 0 PV, which was also the starting point of the EOR stage, show that water flooding resulted in viscous fingering patterns in all cases due to the low viscosity of water compared to oil (Figure 9). However, the flooding patterns of the EOR stage for Case 3 differed from those patterns in Cases 1 and 4. For Case 3, only a small amount of oil in the left bottom area of the microchannel was peeled off by the displacing fluids (API brine + 10% ammonia) from 0 to 1.8 PV, and afterward, the distribution pattern of the oil phase had almost no change thoughout the EOR stage. This phenomenon is common for traditional water flooding in which water flows in a fingerlike pattern due to the viscosity difference between water and oil. The oil recovery efficiency, as a result, is usually low because water breaks though early and bypasses the unswept oil area with low sweep efficiency, as shown by the schematic in Figure 11a.

**Figure 10:**
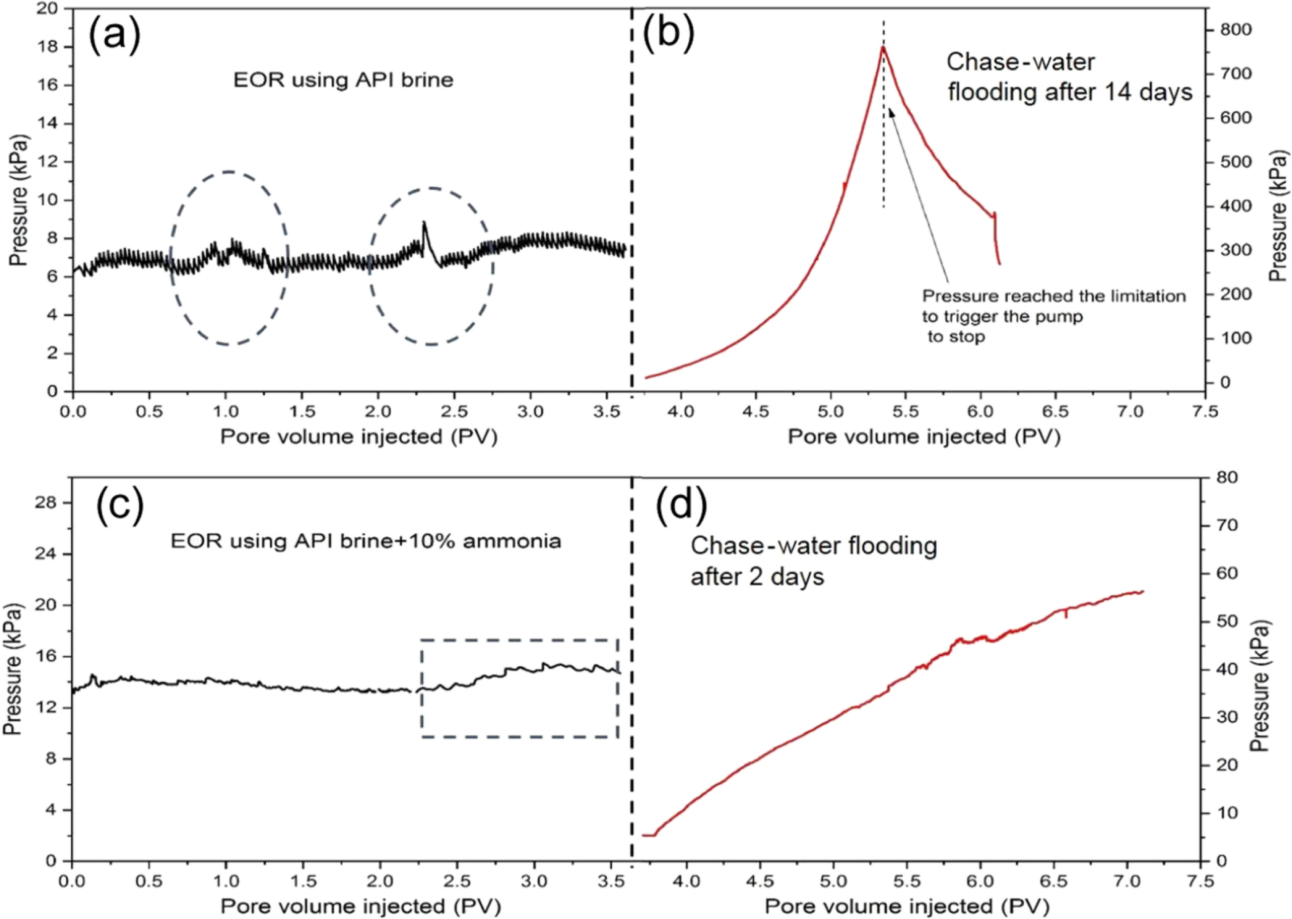
(a) Differential pressure for the EOR stage 1 day after water flooding for Case 1 and (b) 14 days after EOR flooding for Case 1 and (c) differential pressure for the EOR stage 1 day after water flooding for Case 4 and (d) 2 days after EOR flooding for Case 4.

**Figure 11:**
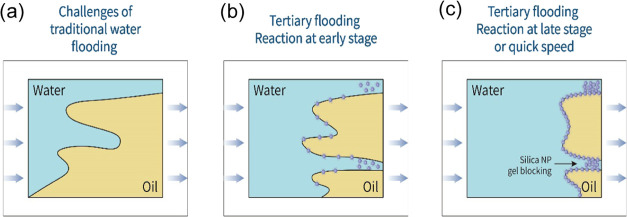
(a) “Thief zone” existing in the traditional water flooding, (b) SiO_2_ assembling on the water–oil interface and diffusing into the aqueous phase, and (c) elastic interface fully covered by particles and the bypass channel blocked by soft gel formed with high-concentration particles.

For Case 1, obvious oil recovery was witnessed within area “*b*” from 0 to 1.8 PV (Figure 9), corresponding to the pressure fluctuations between 0.75 and 1.3 PV shown by the dashed ellipsoid in Figure 10a. However, the oil mobilization was not effective or durable after 1.8 PV because there was no NH_4_OH added to the brine and the production of SiO_2_ nanoparticles was only dependent on the spontaneous hydrolysis of TEOS precursor during the EOR stage, leading to a deficient number of particles assembling at the oil–water interface and consequently a weak influence on the interface properties, as illustrated in Figure 11b.

For Case 4, the oil phase within area “*b*” was gradually wiped away from 0 to 2.25 PV (Figure 9), coinciding with the “climbing-then-descending” pattern of pressure evolution in Figure 10c, which was also the classical pressure trend for flooding experiments in core-scale sandstones.^4^ The pressure will go down theoretically as more oil is continuously removed from 2.25 to 3.5 PV as shown in the area “*b*” for Case 4. However, unlike the classical pressure descending trend, the corresponding pressure line shown by the dashed rectangular square in Figure 10c went up obviously from ∼2.25 PV, which was due to the silica nanoparticle formation. As investigated on the bulk scale in Figure 4a, *in situ* formation and assembly of superfluous nanoparticles at the interface would elasticate the interface, thus hindering the interface from deforming and mobilizing to some extent. This process was also affected by the presence of ammonia, which could increase the speed of interfacial reaction and particle formation, as shown by the schematic diagram in Figure 11c.

The oil in area “*a*” circled by a yellow dashed boundary, which was also the immiscible oil–water interface, was mobilized by flooding fluids for all cases before 1.8 PV in Figure 9. However, after 1.8 PV, the boundary of the previous flow path in Cases 1 and 4 gradually became elastic and it was harder for it to deform because the *in situ* synthesized nanoparticles assembled and jammed at the interface, similar to the analysis of bulk interface properties in Section 3.1. Our previous study has shown that nanoparticles tended to stay at the oil–water interface after synthesis due to the high energy required to detach particles, and bending the interface to expose more nanoparticles to either aqueous or oil phase would be energetically unfavorable.^3^ The boundary of area “*a*” was therefore transformed into a circulated “wall” to close the area previously allowing fluids to pass. The displacing fluid was then diverted to the unswept area “*b*”. This process equivalently introduced a “shut-off” effect in the reservoir.^43,44^

Due to the relatively high concentration of TEOS used and as the reaction proceeded, a large number of nanoparticles modified with organic species could subsequently diffuse into the aqueous brine and behave as a soft gel to further reduce the relative permeability for displacing fluids, as illustrated in Figures 1 and 11c. Therefore, nanoparticle assembly at the immiscible oil–water interface and surface-modified particles enriched in the aqueous brine could synergically increase the flow resistance to the previous water-flowing finger channel and consequently reduce the relative permeability of displacing fluid in the so-called “thief zone”, which is shown as zone “*a*” in Figure 9.

The “shut-off” effect and flow resistance were even more significant though aging after the EOR flooding stage. As shown in Figure 10b, the pressure for Case 1 soared up to the preset limitation of the microfluidic system (800 kPa) after 14 days of aging because the boundary of area “*a*” in Figure 9 was further enhanced by NPs, which were continually formed in 14 days, and plugging of flow path was introduced by the silica gel agglomerated in the brine phase. By contrast, the oil–water interface was more quickly occupied by nanoparticles in Case 4, leading to an earlier shut-off effect for the whole chip in just 2 days of aging. This was because the reaction rate was much faster with the presence of NH_4_OH as a catalyst in Case 4. This was confirmed by the steady pressure increase in Figure 10d for the chase-water flooding only 2 days after the EOR flooding.

It is noteworthy that the precursor and catalyst concentration demonstrated in this research was quite high and unoptimized, and further practical application of this concept for enhanced oil recovery could rely on optimized precursor concentration by comprehensively considering EOR efficiency and better flow assurance. TEOS is dissolvable in the oil phase; therefore, it is possible to preinject some oil containing this reactant into the reservoir, then recover more crude oil, and achieve economic benefits. Furthermore, the reservoir conditions, including crude oil, high temperature, and formation pressure, should be applied in experimental studies from a practical point of view.

## 4 Conclusions

This work conducted a feasibility study of *in situ* producing nanoparticles inside reservoirs for enhanced oil recovery instead of injecting presynthesized particles from outside (*ex situ*). A proof study of silica nanoparticle formation at the oil–water interface by the hydrolyzing method from a single precursor TEOS was performed in this work. First, bulk-scale experiments were conducted to reveal the reaction kinetics and interface properties for the *in situ* reaction process. Then, micromodel experiments were performed to evaluate the oil recovery potential and displacement mechanisms from the pore scale. The micromodel flooding experiments showed that the quick reaction in Case 4 (oil + 30% TEOS, 10% NH_4_OH in brine) achieved an EOR efficiency of 25.4% during the tertiary flooding process, which resulted in a total oil recovery efficiency of 56.7%. In contrast, the slow reaction in Case 1 (oil + 30% TEOS, brine) only mobilized an additional 14.6% oil at the EOR stage due to the absence of a catalytic effect for NP production.

The study also shows that there are two main mechanisms for EOR: wettability alteration and conformance control. The wettability alteration, as a result of nanoparticles’ formation at the oil–water interface and deposition on the microchannel surface, occurred in the early stage of the water flooding stage as long as the precursor was present in the oil phase. The formed nanoparticles could alter the micromodel wettability from strong water wetting toward intermediate wetting. As the tertiary flooding process continued, surface-coated silica nanoparticles were accumulatively produced, and they assembled at the oil–water interface, forming a barrier toward interface deformation, and were also enriched in the aqueous phase to behave as a soft gel. Consequently, the flow resistance in bypass routes caused by water flooding was increased, which yielded a “shut-off” effect and diverted displacing fluid to the unswept zone. The results also suggest that the reaction rate and reactant concentration need to be more controlled for future reservoir applications.
